# Associations of Sedentary Behavior, Sedentary Bouts and Breaks in Sedentary Time with Cardiometabolic Risk in Children with a Family History of Obesity

**DOI:** 10.1371/journal.pone.0079143

**Published:** 2013-11-20

**Authors:** Travis John Saunders, Mark Stephen Tremblay, Marie-Ève Mathieu, Mélanie Henderson, Jennifer O’Loughlin, Angelo Tremblay, Jean-Philippe Chaput, on behalf of the QUALITY cohort research group

**Affiliations:** 1 Healthy Active Living and Obesity Research Group, Children’s Hospital of Eastern Ontario Research Institute, Ottawa, Ontario, Canada; 2 School of Human Kinetics, Faculty of Health Sciences, University of Ottawa, Ottawa, Ontario, Canada; 3 Department of Kinesiology, University of Montreal, Montreal, Quebec, Canada; 4 Department of Pediatrics, CHU Ste-Justine and University of Montreal, Montreal, Quebec, Canada; 5 Department of Social and Preventive Medicine, Faculty of Medicine, University of Montreal, Montreal, Quebec, Canada; 6 Department of Kinesiology, Faculty of Medicine, Laval University, Quebec City, Quebec, Canada; Scientific Directorate, Bambino Hospital, Italy

## Abstract

**Background:**

Although reports in adults suggest that breaks in sedentary time are associated with reduced cardiometabolic risk, these findings have yet to be replicated in children.

**Purpose:**

To investigate whether objectively measured sedentary behavior, sedentary bouts or breaks in sedentary time are independently associated with cardiometabolic risk in a cohort of Canadian children aged 8–11 years with a family history of obesity.

**Methods:**

Data from 286 boys and 236 girls living in Quebec, Canada, with at least one biological parent with obesity (QUALITY cohort) were collected from 2005–2008, and analyzed in 2013. Sedentary behavior, light and moderate-to-vigorous physical activity were measured over 7 days using accelerometry. Leisure time computer/video game use and TV viewing over the past 7 days were self-reported. Outcomes included waist circumference, body mass index Z-score, fasting insulin, fasting glucose, triglycerides, HDL-cholesterol, C-reactive protein and a continuous cardiometabolic risk score.

**Results:**

After adjustment for confounders, breaks in sedentary time and the number of sedentary bouts lasting 1–4 minutes were associated with reduced cardiometabolic risk score and lower BMI Z-score in both sexes (all *p*<0.05). The number of sedentary bouts lasting 5–9 minutes was negatively associated with waist circumference in girls only, while the number of bouts lasting 10–14 minutes was positively associated with fasting glucose in girls, and with BMI Z-score in boys (all *p*<0.05). Leisure time computer/video game use was associated with increased cardiometabolic risk score and waist circumference in boys, while TV viewing was associated with increased cardiometabolic risk, waist circumference, and BMI Z-score in girls (all *p*<0.05).

**Conclusions:**

These results suggest that frequent interruptions in sedentary time are associated with a favourable cardiometabolic risk profile and highlight the deleterious relationship between screen time and cardiometabolic risk among children with a family history of obesity.

## Background

Sedentary behavior (e.g. sitting or reclining while expending ≤1.5 metabolic equivalents) [1] is independently associated with increased cardiometabolic risk in children and youth [2]–[10]. Recent systematic reviews have reported that sedentary behavior is associated with reduced cardiorespiratory fitness, increased adiposity and elevated risk of metabolic syndrome in the pediatric age group [3], [4]. However, while a growing body of evidence suggests that sedentary behavior represents a novel risk factor for chronic disease among children and youth, it is unclear which characteristics and modalities of sedentary behavior are most closely associated with increased health risk in this population [5], [7], [8], [11].

Self-reported screen-based sedentary behaviors (e.g. television viewing, computer use, video game playing, etc.) have been consistently associated with increased markers of cardiometabolic risk in children and youth, independent of physical activity levels [3], [5], [7]–[9]. In contrast, studies examining accelerometer-derived measures of sedentary behavior in this age group have often failed to detect a significant association with markers of cardiometabolic risk after adjustment for confounders [5], [11]–[14]. Similarly, while interruptions in objectively measured sedentary time are beneficially associated with markers of cardiometabolic risk in adults [15], [16], these findings have yet to be replicated in the pediatric age group [5], [11] where activity profiles are highly intermittent [17]. A better understanding of the relationship between characteristics of sedentary behavior and markers of cardiometabolic risk is necessary to inform lifestyle interventions and public health policies aimed at reducing chronic disease risk in children and youth.

The purpose of the present study was to investigate whether objectively measured sedentary time, or characteristics related to the accumulation of sedentary behavior (e.g. breaks in sedentary time or the accumulation of sedentary time in bouts of various lengths) are independently associated with cardiometabolic risk in a cohort of Canadian children aged 8–11 years with a family history of obesity. It was hypothesized that a continous cardiometabolic risk score would be positively associated with sedentary behavior, and negatively associated with breaks in sedentary time in this population.

## Materials and Methods

### Study Population

The sample consisted of 630 children enrolled in the QUebec Adiposity and Lifestyle InvesTigation in Youth (QUALITY) cohort, which has been described previously [18]. Briefly, participants in the QUALITY cohort are white and aged 8–11 years at study entry, and all participants have at least one biological parent with obesity (i.e. a body mass index (BMI) ≥30 kg/m^2^ or abdominal waist circumference ≥88 cm for women or ≥102 cm for men). Children were excluded from the cohort if they were consuming a very low calorie diet (≤600 kcal/day), had a serious physical or mental health condition that could compromise participation in the study, had diabetes (type 1 or type 2), or were currently taking steroids, β-blockers, thiazides or other drugs for hypertension.

Roughly 400 000 flyers were distributed between 2005 and 2008 to families with children in Grades 2–5, in 1040 primary schools within 75 km of Montreal, Quebec City and Sherbrooke in Quebec, Canada. Of 3350 families who contacted the study coordinator, 1320 met all inclusion criteria. Reasons for non-participation at baseline among eligible families were: (i) not interested, 81%; (ii) at least one parent did not agree to participate or was unavailable, 11%; (iii) child declined to participate, 4%; (iv) lived too far from a study centre, 2%; (v) insufficient time, 1%; and (vi) other, 1%.

All data included in the present analysis were collected during baseline examinations between 2005 and 2008. The present cross-sectional analysis was performed in 2013 and includes 522 participants with complete data for all variables of interest.

### Ethics Statement

This project was approved by the institutional ethics review boards at Centre Hospitalier Universitaire Sainte-Justine and Laval University. Written informed parental consent and child assent were obtained for all participants, in accordance with the principles expressed in the Declaration of Helsinki.

### Outcome Measures

All markers of cardiometabolic risk were assessed during a hospital visit. Height was measured to the nearest millimeter using a wall-mounted stadiometer. Weight was assessed to the nearest 0.1 kg using a spring scale that was calibrated daily. Waist circumference was assessed at the midpoint of the lowest rib and iliac crest at the end of a normal exhalation. Body mass index (BMI) was calculated by dividing body mass (kg) by height in meters squared, and converted to a BMI Z-score based on values published by the Centers for Disease Control and Prevention [19]. All anthropometric measurements were taken in duplicate with participants wearing indoor clothing without shoes or sweaters and measured according to standardized methods by trained research assistants [18].

Metabolic markers were assessed using venous blood samples collected following a 12-hour overnight fast, analyzed in batches at a single site (CHU Sainte-Justine Clinical Biochemistry laboratory) [18]. Plasma insulin was measured with the ultrasensitive Access immunoassay system (Beckman Coulter, Brea, CA, USA). Glucose (oxidase method), HDL-C and triglycerides (enzymatic method) were measured using a Synchron LX, while high sensitivity C-Reactive Protein (hs-CRP) (immunoassay method) was measured using a Synchron CX (Beckman Coulter, Brea, CA, USA). Blood pressure was measured on the right arm, with the child in a sitting position and at rest for at least 5 min, using an oscillometric instrument (Dinamap model CR9340, GE Healthcare, Mississauga, ON). Three consecutive measures were obtained with a 1 minute break between each measure. The average value of the 3 measures was used in the present analyses.

### Calculation of a Continuous Cardiometabolic Risk Score

A sex-specific continuous cardiometabolic risk score was calculated for each participant as follows:
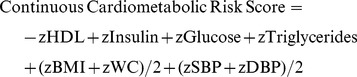



This cardiometabolic risk score was used as a means of estimating an individual’s global cardiometabolic risk. In contrast to a dichotomous metabolic syndrome diagnosis, this approach results in a continuous risk score that increases statistical power, and has been used in several recent investigations in the pediatric population [6], [20], [21].

### Physical Activity and Sedentary Behavior

Objectively measured sedentary behavior and physical activity were assessed using the Actigraph LS 7164 accelerometer (Actigraph, Pensacola, FL, USA) for one week. Participants were instructed to wear the accelerometer on the right hip during all waking hours, except during bathing or aquatic activities such as swimming. Acclerometry data were downloaded as 1-min epochs and were processed using SAS 9.2 (SAS Institute, Cary, NC, USA) and Microsoft Excel (Microsoft, Redmond, WA, USA) according to standardized quality control and data reduction procedures [22]. Non-wear time was defined as at least 60 consecutive minutes of zero counts, with allowance for up to 2 minutes of counts between 0 and 100 [22]. A valid day was defined as ≥10 hours of monitor wear time, and only participants with 4 or more valid days (including at least one weekend day) were included in the present analyses. There were no significant differences in any marker of cardiometabolic risk between participants with and without valid accelerometer data (data not shown).

Sedentary behavior was defined as all minutes with an average activity count of less than 100 counts/minute, light physical activity (LPA) as all minutes with an activity count of 100–2296 counts/minute, and moderate-to-vigorous intensity physical activity (MVPA) as any minute with an activity count greater than 2296 counts/minute [23]. A sedentary bout was defined as 1 or more consecutive minutes with less than 100 counts/minute. The number of daily bouts of sedentary time lasting 1–4 minutes, 5–9 minutes, 10–14 minutes, 15–29 minutes, and 30+ minutes were calculated for each participant. Breaks in sedentary time were calculated as any interruption in sedentary time lasting one minute or longer in which the accelerometer counts per minute rose up to or above 100 [15]. Daily television (TV) viewing, and leisure time computer/video game use (surfing the internet, playing video games on a computer or other device, etc.) were assessed using self-report questionnaires. Participants were asked how many hours they spent watching TV and using the computer for fun on weekdays and weekend days, and a mean score over the 7 days was computed. These questions are similar to those used in the Youth Risk Behavior Survey, and have been shown to be valid and reliable in the pediatric age group [24].

### Covariates

Sexual maturation was assessed by a research nurse and was scored from 1 (pre-pubertal) to 5 (adult) according to Tanner stages [25], [26]. Ten percent of boys and 35% of girls had a Tanner stage of 2 or higher, indicating that they had begun puberty. Baseline questionnaires ascertained highest educational level of the parents (high school, pre-university level [Collège d’enseignement général et professionnel for Quebec], university) and total annual family income (categorized into 12 groups ranging from <$10,000 to $140,000 CAD or more).

### Statistical Analyses

Sex-by-sedentary behavior interactions were investigated for all outcomes of interest. Significant sex interactions were observed for waist circumference, BMI Z-score, glucose, insulin, and hs-CRP, therefore all analyses have been performed in boys and girls separately. Fasting insulin and plasma triglycerides were non-normally distributed and were therefore transformed using a Box-Cox transformation prior to their inclusion in statistical analyses.

Independent t-tests were performed to assess differences in behavioral and cardiometabolic risk factors between boys and girls. Simple correlations were used to examine the relationship between self-reported and accelerometer-derived sedentary behavior. Regression analyses were performed to determine the associations between sedentary behavior and both the continuous cardiometabolic risk score and individual markers of cardiometabolic risk. Initial models were unadjusted, while subsequent analyses adjusted for accelerometer wear time, age, light and moderate-to-vigorous physical activity, total sedentary time, BMI Z-score (unless included in the outcome), Tanner stage, parental income and level of education. These covariates were chosen as they were associated with multiple markers of cardiometabolic risk in both sexes (all p<0.05). Statistical significance was set at a p value of <0.05. All statistical analyses were performed using SAS version 9.2 (SAS Institute, Cary, NC).

## Results

Characteristics of study participants are presented in Table 1. In comparison to girls, boys were significantly more physically active and spent more time using computers/playing video games in their leisure time (all p<0.01). Boys also had higher concentrations of fasting glucose and HDL-Cholesterol and lower diastolic blood pressure, triglycerides and fasting insulin (all p<0.01). There were no differences between boys and girls in age, objectively measured sedentary time, LPA, self-reported television viewing, continuous cardiometabolic risk score or any anthropometric measurement (all p>0.05). The number of daily sedentary bouts of each length was similar for both sexes. Boys accumulated fewer bouts of sedentary behavior lasting 1–4 minutes (p<0.05) while there were no differences between sexes for the number of sedentary bouts lasting 5–9 minutes, 15–29 minutes, or 30+ minutes (all p>0.05). Accelerometer-derived sedentary time was positively associated with leisure time computer/video game use in boys only (r = 0.20, p = 0.008), but was not associated with self-reported TV viewing in either sex (all p>0.10).

**Table 1:** Characteristics of study participants.

	Boy (n = 286)	Girl (n = 236)	*P value*
Age (years)	9.2 (9.1, 9.3)	9.1 (9.0, 9.2)	0.55
Height (cm)	139.3 (138.4, 140.2)	138.4 (137.3, 139.5)	0.20
Weight (kg)	38.2 (36.9, 39.5)	38.1 (36.7, 39.6)	0.94
BMI (kg/m^2^)	19.4 (18.9, 19.9)	19.6 (19.0, 20.1)	0.61
Waist Circumference (cm)	67.6 (66.2, 69.0)	67.3 (65.8, 68.8)	0.82
Sedentary Time (min/day)	363.5 (354.9, 372.1)	366.7 (358.1, 375.4)	0.61
Number of valid days of accelerometry (days)	6.5 (6.4, 6.6)	6.5 (6.4, 6.6)	0.98
Number of hours of accelerometry data (hours/day)	13.8 (13.7, 13.9)	13.6 (13.5, 13.7)	0.02
LPA (min/day)	403.9 (397.1, 410.6)	409.5 (402.9, 416.1)	0.24
MVPA (min/day)	61.2 (57.8, 64.6)	41.2 (38.8, 43.6)	<0.01
Sedentary Bouts 1–4 Minutes (number/day)	67 (66, 68)	70 (69, 72)	<.01
Sedentary Bouts 5–9 Minutes (number/day)	13 (12, 14)	13 (13, 14)	0.58
Sedentary Bouts 10–14 Minutes (number/day)	4 (4, 5)	4 (4, 5)	0.88
Sedentary Bouts 15–29 Minutes (number/day)	3 (3, 3)	3 (3, 3)	0.92
Sedentary Bouts 30+ Minutes (number/day)	2 (2, 2)	2 (2, 2)	0.22
TV viewing (hours/day)	2.0 (1.8, 2.2)	1.8 (1.6, 2.0)	0.12
Computer/video game use (hours/day)	1.1 (0.9, 1.2)	0.6 (0.5, 0.7)	<0.01
Systolic BP (mmHg)	95 (94, 96)	94 (93, 95)	0.23
Diastolic BP (mmHg)	49 (49, 50)	50 (50, 51)	0.01
Insulin (pmol/L)	30.1 (27.9, 32.3)	38.2 (34.9, 41.5)	<0.01
Glucose (mmol/L)	5.00 (4.96, 5.04)	4.90 (4.85, 4.94)	<0.01
HDL-Cholesterol (mmol/L)	1.22 (1.19, 1.25)	1.16 (1.13, 1.19)	<0.01
Triglycerides (mmol/L)	0.76 (0.72, 0.80)	0.89 (0.84, 0.95)	<0.01
hs-CRP (mg/L)	1.09 (0.82, 1.36)	1.20 (0.92, 1.48)	0.57
Continuous Cardiometabolic Risk Score	0.03 (−0.41, 0.48)	0.03 (−0.47, 0.53)	0.98

Data presented as means (95% confidence intervals).

P values represent sex differences assessed using an independent Student’s t-test.

Continuous cardiometabolic risk score was calculated by summing z-scores for insulin, glucose, triglycerides, negative HDL-cholesterol, blood pressure, BMI, and waist circumference for each participant.

BMI, body mass index; LPA, light intensity physical activity; MVPA, moderate-to-vigorous physical activity; TV, television; BP, blood pressure; HDL, high density lipoprotein; hs-CRP, high sensitivity C-reactive protein.

### Unadjusted Associations

Associations between characteristics of sedentary behavior and markers of cardiometabolic disease risk are presented in Tables 2 and 3. In boys, the continuous cardiometabolic risk score was positively associated with total sedentary time, the number of sedentary bouts lasting 10–14 minutes, the number of bouts lasting 15–29 minutes, and both TV viewing and leisure time computer/video game use, while it was negatively associated with the number of sedentary bouts lasting 1–4 minutes (all p<0.05). Among girls, the continuous cardiometabolic risk score was positively associated with total sedentary time, sedentary bouts lasting 5–9, 10–14 minutes, and 15–29 minutes, as well as both TV viewing and leisure time computer/video game use (all p<0.05).

**Table 2:** Associations of sedentary behavior with markers of cardiometabolic risk in boys.

	Continuous Cardiometabolic Risk	WC (cm)	BMI (Z-Score)	Insulin (pmol/L)	Glucose (mmol/L)	HDL-C (mmol/L)	Triglycerides (mmol/L)	hs-CRP (mg/L)
*Model 1*								
Sedentary Time (min/day)	0.010 (0.004, 0.016)*	0.040 (0.022, 0.059)*	0.002 (0.001, 0.004)*	0.001 (0.001, 0.002)*	0.0001 (−0.0004, 0.001)	−0.0003 (−0.0007, 0.0001)	0.001 (−0.0002, 0.001)	0.004 (0.001, 0.006)*
Breaks in Sedentary Time (number/day)	−0.020 (−0.059, 0.018)	−0.093 (−0.215, 0.030)	−0.006 (−0.017, 0.004)	−0.004 (−0.010, 0.001)	−0.002 (−0.005, 0.002)	−0.001 (−0.004, 0.001)	−0.004 (−0.008, 0.001)	0.002 (−0.014, 0.019)
Sedentary Bouts 1–4 Minutes (number/day)	−0.065 (−0.100, −0.003)*	−0.271 (−0.82, −0.161)*	−0.019 (−0.028, −0.009)*	−0.009 (−0.014, −0.005)*	−0.002 (−0.005, 0.002)	0.001 (−0.002, 0.003)	−0.005 (−0.010, −0.001)*	−0.017 (−0.032, −0.002)*
Sedentary Bouts 5–9 Minutes (number/day)	0.069 (−0.076, 0.214)	0.357 (−0.103, 0.816)	0.010 (−0.028, 0.049)	0.006 (−0.015, 0.026)	−0.004 (−0.018, 0.009)	−0.008 (−0.018, 0.002)	0.003 (−0.015, 0.021)	0.077 (0.014, 0.139)*
Sedentary Bouts 10–14 Minutes (number/day)	0.594 (0.279, 0.908)*	2.526 (1.547, 3.506)*	0.158 (0.074, 0.241)*	0.078 (0.034, 0.121)*	−0.010 (−0.041, 0.020)	−0.026 (−0.048, −0.004)*	0.038 (−0.001, 0.076)	0.219 (0.084, 0.354)*
Sedentary Bouts 15–29 Minutes (number/day)	0.391 (0.059, 0.723)*	2.071 (1.034, 3.107)*	0.122 (0.034, 0.210)*	0.063 (0.017, 0.108)*	0.006 (−0.025, 0.038)	−0.004 (−0.026, 0.019)	0.006 (−0.035, 0.047)	0.126 (−0.016, 0.269)
Sedentary Bouts 30+ Minutes (number/day)	0.6620 (−0.015, 1.256)	2.973 (0.962, 4.984)*	0.217 (0.048, 0.386)*	0.109 (0.021, 0.197)*	−0.006 (−0.065, 0.053)	−0.012 (−0.056, 0.031)	0.049 (−0.029, 0.127)	0.066 (−0.209, 0.341)
TV Viewing (hours/day)	0.465 (0.204, 0.726)*	0.904 (0.068, 1.740)*	0.088 (0.017, 0.160)*	0.041 (0.005, 0.078)*	0.024 (−0.0001, 0.049)	−0.015 (−0.033, 0.003)	0.027 (−0.006, 0.060)	0.130 (0.017, 0.243)*
Computer/Video Game Use (hours/day)	0.687 (0.300, 1.073)*	1.629 (0.394, 2.863)*	0.058 (−0.049, 0.164)	0.066 (0.012, 0.119)*	0.032 (−0.004, 0.068)	−0.043 (−0.069, −0.017)*	0.028 (−0.021, 0.076)	0.173 (0.007, 0.340)*
*Model 2*								
Sedentary Time (min/day)	0.011 (−0.019, 0.041)	0.038 (−0.012, 0.087)	−0.001 (−0.009, 0.008)	0.001 (−0.003, 0.004)	0.002 (−0.001, 0.005)	−0.001 (−0.003, 0.001)	−0.0004 (−0.004, 0.003)	0.003 (−0.009, 0.015)
Breaks in Sedentary Time (number/day)	−0.057 (−0.106, −0.008)*	−0.027 (−0.110, 0.057)	−0.026 (−0.040, −0.012)*	−0.002 (−0.008, 0.004)	0.001 (−0.005, 0.006)	−0.001 (−0.005, 0.002)	−0.005 (−0.011, 0.001)	0.005 (−0.014, 0.025)
Sedentary Bouts 1–4 Minutes (number/day)	−0.063 (−0.111, −0.015)*	−0.052 (−0.133, 0.030)	−0.028 (−0.041−0.016)*	−0.001 (−0.007, 0.005)	−0.001 (−0.006, 0.004)	−0.001 (−0.004, 0.003)	−0.002 (−0.008, 0.004)	−0.00002 (−0.019, 0.019)
Sedentary Bouts 5–9 Minutes (number/day)	−0.048 (−0.245, 0.148)	0.080 (−0.244, 0.404)	−0.039 (−0.094, 0.015)	−0.007 (−0.030, 0.017)	−0.005 (−0.025, 0.015)	−0.011 (−0.025, 0.003)	0.005 (−0.019, 0.029)	0.047 (−0.029, 0.123)
Sedentary Bouts 10–14 Minutes (number/day)	0.473 (−0.006, 0.952)	0.334 (−0.468, 1.135)	0.169 (0.035, 0.302)*	0.001 (−0.057, 0.060)	−0.041 (−0.091, 0.009)	−0.030 (−0.065, 0.004)	0.018 (−0.041, 0.077)	−0.073 (−0.262, 0.115)
Sedentary Bouts 15–29 Minutes (number/day)	−0.165 (−–0.735, 0.405)	−0.721 (−1.653, 0.211)	0.128 (−0.029, 0.285)	−0.033 (−0.101, 0.035)	−0.004 (−0.063, 0.055)	0.026 (−0.015, 0.067)	−0.072 (−0.140, −0.003)*	−0.279 (−0.498, −0.060)*
Sedentary Bouts 30+ Minutes (number/day)	0.321 (−0.411, 1.054)	0.194 (−1.021, 1.409)	0.153 (−0.051, 0.357)	0.038 (−0.050, 0.126)	0.004 (−0.070, 0.077)	0.007 (−0.046, 0.060)	0.017 (−0.072, 0.106)	−0.171 (−0.447, 0.106)
TV Viewing (hours/day)	0.249 (−0.020, 0.519)	−0.176 (−0.623, 0.272)	0.050 (−0.026, 0.125)	0.003 (−0.029, 0.036)	0.020 (−0.008, 0.048)	−0.001 (−0.021, 0.018)	−0.006 (−0.039, 0.027)	0.050 (−0.055, 0.155)
Computer/Video Game Use (hours/day)	0.485 (0.084, 0.886)*	0.799 (0.141, 1.457)*	0.016 (−0.097, 0.128)	0.031 (−0.017, 0.079)	0.013 (−0.028, 0.055)	−0.041 (−0.070 −0.012)*	0.015 (−0.034, 0.064)	0.145 (−0.011, 0.300)

Model 1. Unadjusted analyses.

Model 2. Adjusted for accelerometer wear time, age, light and moderate-to-vigorous physical activity, total sedentary time (except when exposure), BMI Z-score (except when included in outcome), Tanner stage, parental income and level of education. Data are presented as beta coefficients (95% confidence intervals). n = 286.

Associations assessed using linear regression analysis. Data are presented as beta coefficients (95% confidence intervals). n = 286.

* = p<0.05.

Fasting insulin and plasma triglycerides have been transformed using a Box-Cox transformation.

Continuous cardiometabolic risk score was calculated by summing z-scores for insulin, glucose, triglycerides, negative HDL-cholesterol, blood pressure, BMI, and waist circumference for each participant.

BMI, body mass index; HDL-C, high density lipoprotein cholesterol; hs-CRP, high sensitivity c-reactive protein; MVPA, moderate-to-vigorous physical activity; TV, television; WC, waist circumference.

**Table 3:** Associations of sedentary behavior with markers of cardiometabolic risk in girls.

	Continuous Cardiometabolic Risk	WC (cm)	BMI (Z-score)	Insulin (pmol/L)	Glucose (mmol/L)	HDL-C (mmol/L)	Triglycerides (mmol/L)	hs-CRP (mg/L)
*Model 1*								
Sedentary Time (min/day)	0.010 (0.002, 0.017)*	0.034 (0.013, 0.056)*	0.001 (−0.001, 0.003)	0.020 (0.001, 0.003)*	0.001 (−0.00004, 0.0010)	−0.0002 (−0.001, 0.0002)	0.0001 (−0.001, 0.001)	−0.001 (−0.004, 0.002)
Breaks in Sedentary Time(number/day)	0.013 (−0.039, 0.065)	−0.020 (−0.177, 0.137)	−0.009 (−0.023, 0.005)	0.004 (−0.004, 0.012)	0.004 (−0.001, 0.009)	0.001 (−0.002, 0.004)	−0.00004 (−0.006, 0.006)	−0.009 (−0.030, 0.011)
Sedentary Bouts 1–4 Minutes(number/day)	−0.016 (−0.064, 0.032)	−0.124 (−0.266, 0.017)	−0.009 (−0.022, 0.004)	−0.004 (−0.011, 0.004)	−0.00001 (−0.004, 0.004)	0.001 (−0.002, 0.004)	0.0004 (−0.005, 0.006)	−0.005 (−0.024, 0.014)
Sedentary Bouts 5–9 Minutes(number/day)	0.211 (0.031, 0.391)*	0.527 (−0.009, 1.063)	0.024 (−0.026, 0.074)	0.043 (0.016, 0.071)*	0.012 (−0.004, 0.029)	−0.004 (−0.015, 0.007)	0.001 (−0.021, 0.022)	0.013 (−0.058, 0.085)
Sedentary Bouts 10–14 Minutes(number/day)	0.510 (0.120, 0.899)*	1.025 (−0.141, 2.191)	−0.016 (−0.124, 0.092)	0.087 (0.027, 0.146)*	0.052 (0.017, 0.087)*	−0.016 (−0.039, 0.008)	0.012 (−0.034, 0.058)	−0.057 (−0.211, 0.097)
Sedentary Bouts 15–29 Minutes(number/day)	0.413 (0.012, 0.814)*	1.512 (0.338, 2.686)*	0.017 (−0.093, 0.126)	0.086 (0.025, 0.146)*	0.026 (−0.010, 0.062)	−0.012 (−0.036, 0.012)	−0.006 (−0.053, 0.040)	−0.048 (−0.206, 0.110)
Sedentary Bouts 30+ Minutes(number/day)	0.442 (−0.314, 1.198)	1.346 (−0.906, 3.597)	0.033 (−0.174, 0.241)	0.130 (0.015, 0.246)*	0.012 (−0.056, 0.080)	0.014 (−0.032, 0.059)	0.044 (−0.044, 0.133)	−0.248 (−0.544, 0.047)
TV Viewing (hours/day)	0.774 (0.475, 1.072)*	2.458 (1.557, 3.359)*	0.176 (0.090, 0.261)*	0.099 (0.053, 0.145)*	0.023 (−0.005, 0.051)	−0.030 (−0.048, −0.011)*	0.050 (0.014, 0.087)*	0.196 (0.074, 0.319)*
Computer/Video Game Use (hours/day)	0.902 (0.314, 1.490)*	3.215 (1.456, 4.974)*	0.191 (0.026, 0.356)*	0.133 (0.044, 0.222)*	0.010 (−0.043, 0.064)	−0.037 (−0.073, −0.001)*	0.058 (−0.011, 0.127)	0.203 (−0.033, 0.440)
*Model 2*								
Sedentary Time (min/day)	0.014 (−0.353, 0.957)	−0.015 (−0.056, 0.027)	0.005 (−0.003, 0.014)	−0.0001 (−0.003, 0.003)	−0.0001 (−0.003, 0.003)	−0.0001 (−0.002, 0.002)	0.001 (−0.003, 0.004)	−0.006 (−0.016, 0.005)
Breaks in Sedentary Time(number/day)	−0.084 (−0.143, −0.024)*	−0.012 (−0.102, 0.079)	−0.032 (−0.049, −0.015)*	−0.001 (−0.008, 0.007)	0.002 (−0.004, 0.008)	0.0001 (−0.003, 0.004)	−0.002 (−0.010, 0.005)	0.009 (−0.014, 0.031)
Sedentary Bouts 1–4 Minutes(number/day)	−0.097 (−0.153, −0.041)*	−0.066 (−0.152, 0.019)	−0.032 (−0.048, −0.016)*	−0.004 (−0.010, 0.003)	−0.001 (−0.006, 0.005)	−0.0003 (−0.004, 0.003)	−0.003 (−0.010, 0.004)	0.008 (−0.013, 0.029)
Sedentary Bouts 5–9 Minutes (number/day)	0.041 (−0.192, 0.274)	−0.355 (−0.686, −0.025)*	0.020 (−0.047, 0.087)	0.004 (−0.023, 0.031)	0.002 (−0.020, 0.024)	0.004 (−0.010, 0.018)	−0.009 (−0.036, 0.018)	0.012 (−0.072, 0.096)
Sedentary Bouts 10–14 Minutes(number/day)	0.484 (−0.106, 1.073)	−0.603 (−1.451, 0.245)	−0.010 (−0.181, 0.162)	0.017 (−0.053, 0.086)	0.078 (0.024, 0.133)*	−0.026 (−0.062, 0.009)	0.049 (−0.020, 0.118)	−0.017 (−0.230, 0.196)
Sedentary Bouts 15–29 Minutes(number/day)	0.512 (−0.209, 1.233)	−0.073 (−1.107, 0.962)	0.157 (−0.049, 0.363)	−0.028 (−0.112, 0.056)	−0.001 (−0.069, 0.067)	−0.020 (−0.064, 0.023)	0.012 (−0.071, 0.096)	−0.067 (−0.328, 0.194)
Sedentary Bouts 30+ Minutes(number/day)	−0.260 (−1.112, 0.592)	0.241 (−0.980, 1.463)	−0.061 (−0.308, 0.185)	0.043 (−0.057, 0.144)	−0.032 (−0.111, 0.046)	0.043 (−0.008, 0.093)	0.062 (−0.038, 0.162)	−0.240 (−0.542, 0.062)
TV Viewing (hours/day)	0.736 (0.404, 1.068)*	0.664 (0.153, 1.174)*	0.197 (0.099, 0.294)*	0.016 (−0.026, 0.058)	0.005 (−0.028, 0.039)	−0.009 (−0.030 0.013)	0.030 (−0.012, 0.072)	0.108 (−0.020, 0.235)
Computer/Video Game Use(hours/day)	0.560 (−0.076, 1.197)	0.548 (−0.373, 1.468)	0.141 (−0.043, 0.325)	0.021 (−0.054, 0.096)	0.006 (−0.055, 0.066)	−0.025 (−0.064, 0.013)	0.020 (−0.055, 0.095)	0.105 (−0.126, 0.335)

Model 1. Unadjusted analyses.

Model 2. Adjusted for accelerometer wear time, age, light and moderate-to-vigorous physical activity, total sedentary time (except when exposure), BMI Z-score (except when included in outcome), Tanner stage, parental income and level of education. Data are presented as beta coefficients (95% confidence intervals). n = 286.

Associations assessed using linear regression analysis.

Associations assessed using linear regression analysis. Data are presented as beta coefficients (95% confidence intervals). n = 236.

* = p<0.05.

Fasting insulin and plasma triglycerides have been transformed using a Box-Cox transformation.

Continuous cardiometabolic risk score was calculated by summing z-scores for insulin, glucose, triglycerides, negative HDL-cholesterol, blood pressure, BMI, and waist circumference for each participant.

BMI, body mass index; HDL-C, high density lipoprotein cholesterol; hs-CRP, high sensitivity c-reactive protein; MVPA, moderate-to-vigorous physical activity; TV, television; WC, waist circumference.

### Adjusted Associations

In the fully adjusted model, breaks in sedentary time were negatively associated with the continuous cardiometabolic risk score (boys: β = −0.057, 95% CI = −0.106, −0.008; girls: β = −0.084, 95% CI = −0.143, −0.024) and BMI Z-scores (boys: β = −0.026, 95% CI = −0.040, −0.012; girls: β = −0.032, 95% CI = −0.048, −0.016) in both sexes (all p<0.05). Similar associations were also observed for the number of sedentary bouts lasting 1–4 minutes. The number of sedentary bouts lasting 5–9 minutes was negatively associated with waist circumference in girls only (β = −0.355, 95% CI = −0.686, −0.025) (p<0.05). The number of sedentary bouts lasting 10–14 minutes was positively associated with fasting glucose in girls (β = 0.078, 95% CI = 0.024, 0.133), and with BMI Z-score in boys (β = 0.169, 95% CI = 0.035, 0.302). The number of sedentary bouts lasting 15–29 minutes was negatively associated with fasting triglycerides (β = −0.072, 95% CI = −0.140, −0.003) and hs-CRP (β = −0.279, 95% CI = −0.498, −0.060) in boys only (all p<0.05). Finally, leisure time computer/video game use was positively associated with continuous cardiometabolic risk (β = 0.485, 95% CI = 0.084, 0.886) and waist circumference (β = 0.799, 95% CI = 0.141, 1.457), and negatively associated with HDL-cholesterol (β = −0.041, 95% CI = −0.070, −0.012) in boys only, while TV viewing was positively associated with continuous cardiometabolic risk (β = 0.736, 95% CI = 0.404, 1.068), waist circumference (β = 0.664, 95% CI = 0.153, 1.174) and BMI Z-score in girls only (β = 0.197, 95% CI = 0.099, 0.294) (all p<0.05).

## Discussion

The results of the present study demonstrate that breaks in sedentary time and short bouts of sedentary behavior (e.g. those lasting 1–4 minutes) are associated with reduced cardiometabolic risk and BMI Z-scores in children aged 8–11 independent of total sedentary time and physical activity. These cross-sectional results suggest that children who frequently interrupt their sedentary time may experience lower levels of cardiometabolic risk than those who accumulate sedentary behavior with less frequent interruptions. Markers of cardiometabolic risk were also more closely associated with self-reported leisure time computer/video game use and TV viewing than with objectively measured total sedentary time in this population.

To our knowledge, this is the first study to report a beneficial association between breaks in sedentary time and global cardiometabolic risk in the pediatric population. Healy and colleagues have previously reported that breaks in sedentary time are independently and beneficially associated with adiposity, glucose metabolism, triglyceride levels and hs-CRP in adults [15], [16] although recent studies have generally failed to detect similar associations in children and youth [5], [11]. Carson and Janssen [5] did not observe any association between breaks in sedentary time and continuous cardiometabolic risk in a representative sample of American children and youth aged 6–19 years. Examining another representative sample Canadian youth aged 6–19 years, Colley et al. [11] found that breaks in sedentary time accumulated after 3 pm on weekdays were associated with lower waist circumference in boys aged 11–14 years. However, they reported that breaks in sedentary time were not significantly associated with any other outcome in older or younger boys, or in girls of any age.

The explanation for this discrepancy between the present findings and previous investigations in the pediatric age group is not immediately clear. While the present analysis focused on children with a parental history of obesity, previous investigations into the role of breaks in sedentary behavior among children and youth have focused on representative samples of the Canadian [11] and American [5] pediatric populations. Due to differences in study methodology (e.g. participant age range, accelerometer model, etc) it is not possible to directly compare levels of overweight/obesity, markers of cardiometabolic disease risk or MVPA across the three studies. However, it is possible that associations between breaks in sedentary time and cardiometabolic risk may be stronger in the present population with a family history of obesity, as parental obesity has been associated with increased childhood cardiometabolic risk by some [27]–[29] but not all studies [30]. This difference in study population may help to explain why the present results are more similar to those reported previously by Healy and colleagues in adults [15], [16], rather than other investigations in children and youth [5], [11].

Several mechanisms have been proposed which could explain the beneficial associations between breaks in sedentary time, short bouts of sedentary time, and continuous cardiometabolic risk observed in the present study. Imposed bouts of prolonged sedentary behavior have been shown to acutely reduce insulin sensitivity and increase triglyceride levels in adults [31], effects which are likely due to reductions in lipoprotein lipase and glucose transport protein activity in skeletal muscle [32], [33]. Similarly, frequent walk breaks have been shown to greatly reduce the acute metabolic impact of prolonged sitting in overweight adults [34]. If the impact of chronic breaks in sedentary time are similar to those observed acutely in adults, this could provide a plausible mechanism linking frequent interruptions in sedentary behavior with lower levels of cardiometabolic disease risk. However, a recent study by Saunders and colleagues failed to detect any acute impact of prolonged sitting, with or without interruptions, on markers of cardiometabolic risk in healthy children and youth [35]. Therefore, given that breaks and short bouts of sedentary behavior were not independently associated with any individual markers of cardiometabolic risk other than BMI Z-score in the present study, it is also possible that excess body weight may simply predispose children toward less frequent interruptions in sedentary time.

The current finding that cardiometabolic risk appears to be more closely associated with self-reported TV viewing and leisure time computer/video game use than with objectively measured sedentary time is consistent with other findings in the pediatric population [5]. As noted recently by Pereira and Power, self-reported sedentary behaviours are poorly understood at present [36]. As a result, the reason for the discrepancy between objective and subjective measures of sedentary behavior in the present study is not clear. Given that self-report measures often differ dramatically from those based on accelerometry [37], [38], it is somewhat surprising that it is self-reported sedentary behaviors which are more consistently associated with health risk in the pediatric population. However, it should be noted that self-reported screen time is only able to assess a single form of sedentary behaviour, while accelerometry provides a global measure of time spent sitting. As noted elsewhere, the two measures are therefore assessing different constructs [39], [40]. This point is underscored by the recent findings of Carson and Janssen, who reported a correlation of just 0.08 between self-reported TV viewing and objectively measured sedentary time in a large sample of American children and youth [5].

The present findings suggest that it may be the behaviors children engage in while seated (e.g. increased food intake), rather than the act of sitting *per se*, that most strongly influences the development of cardiometabolic risk in the pediatric age group [39], [41]–[44]. For example, it has been reported that exposure to both video games [44] and television commercials [43] result in increased *ad libitum* food intake in children and youth. In contrast, sitting passively appears to have no impact on subsequent food intake or other forms of behavioural compensation [39], [43]–[45]. The relationship between screen-based sedentary behaviours and excess food intake may therefore help to explain the associations observed between TV viewing, leisure time computer/video game use, and markers of cardiometabolic disease risk in the present study. More research into the mechanisms linking self-reported and directly measured sedentary behavior with markers of cardiometabolic risk is clearly warranted.

It is interesting to note that the associations between both self-reported and objectively measured aspects of sedentary behaviour appear to be more closely associated with measures of adiposity than with other markers of cardiometabolic disease risk in the present sample. This may be due to the fact that excess adiposity typically precludes the development of cardiometabolic dysfunction in children and youth [46]. For example, it has been reported that just 4% of obese adolescents have type 2 diabetes, whereas greater than 90% of youth with diabetes are overweight or obese [46]. Furthermore, it is known that the duration of obesity is strongly related to the risk of cardiometabolic dysfunction [47]. This may help to explain why sedentary behaviours are consistently associated with diabetes and cardiovascular disease in adults [48], despite the relatively few significant associations observed for markers of cardiometabolic disease risk in the present study.

The present study includes several strengths and limitations that warrant mention. The present study included objectively measured sedentary time and cardiometabolic risk factors. However, it was also cross-sectional in nature, precluding the determination of causality. Screen-based sedentary behaviours were assessed via self-report, which may have introduced additional error into the current analyses, when compared with more objective measures. Self-report measures have been shown to systematically over-estimate physical activity in children and youth [38], and it is possible that screen-based sedentary behaviours may be similarly over- or underestimated in this population. However, it should be noted that any error or response bias would be likely to bias the associations between screen-based sedentary behaviours and markers of cardiometabolic disease risk towards the null, which underscores the associations observed in the present analyses. It should also be noted that the accelerometer protocol employed by the present study may have resulted in some light physical activities (e.g. standing still) being inadvertently identified as sedentary behavior. Future studies which employ inclinometers may therefore be able to more accurately distinguish between seated and standing activities [49]. These findings are also based on a sample of white youth with a family history of obesity, and therefore may not generalize to all children or to other age groups.

## Conclusions

The results of the present study demonstrate that breaks in sedentary time and short bouts of sedentary behavior are independently and beneficially associated with markers of cardiometabolic risk in children with a family history of obesity. These results also suggest that cardiometabolic risk is more closely associated with measures of self-reported leisure time screen time than with objectively measured sedentary time in this population. Future studies should investigate whether minimizing screen time or introducing frequent interruptions in sedentary time prevent the development of cardiometabolic risk among children with a family history of obesity.
